# A Multidisciplinary Strategy for Managing Lower Gastrointestinal Endoscopy Waiting Lists Within the ULSS 3 Serenissima Health Authority in the Venice Metropolitan Area

**DOI:** 10.3390/healthcare14162499

**Published:** 2026-08-12

**Authors:** Marta Soave, Giovanni Marchese, Nicola Veronese, Annalisa Baldan, Domenico Bagnara, Sara Antoniazzi, Federico Cavallari, Francesco Bortoluzzi, Chiara Bovo, Giovanni Carretta, Stefano De Boni, Andrea Cozza, Stefano Vianello

**Affiliations:** 1 Primary Care Department, Azienda ULSS 3 (Unità Locale Socio Sanitaria) 3 “Serenissima”, Mirano-Dolo District, 30174 Venice, Italy; marta.soave@aulss3.veneto.it (M.S.); giovanni.marchese@aulss3.veneto.it (G.M.); 2 Sanitary Direction, Azienda ULSS 3 (Unità Locale Socio Sanitaria) 3 “Serenissima”, 30174 Venice, Italy; annalisa.baldan@aulss3.veneto.it (A.B.); domenico.bagnara@aulss3.veneto.it (D.B.); chiara.bovo@aulss3.veneto.it (C.B.); 3 Gastroenterology Unit, Azienda ULSS 3 (Unità Locale Socio Sanitaria) 3 “Serenissima”, Dolo Hospital, 30174 Venice, Italy; sara.antoniazzi@aulss3.veneto.it; 4 Surgery Unit, Azienda ULSS 3 (Unità Locale Socio Sanitaria) 3 “Serenissima”, Mirano Hospital, 30174 Venice, Italy; federico.cavallari@aulss3.veneto.it; 5 Surgery Unit, Azienda ULSS 3 (Unità Locale Socio Sanitaria) 3 “Serenissima”, Venice Hospital, 30174 Venice, Italy; francesco.bortoluzzi@aulss3.veneto.it; 6 Sanitary Direction, Azienda ULSS 7 (Unità Locale Socio Sanitaria) “Pedemontana”, 36061 Bassano del Grappa, Italy; giovanni.carretta@aulss7.veneto.it; 7 Gastroenterology Unit, Azienda ULSS 3 (Unità Locale Socio Sanitaria) 3 “Serenissima”, Chioggia Hospital, 30174 Venice, Italy; stefano.deboni@aulss3.veneto.it; 8 Department of Cardiac, Thoracic, Vascular Sciences, and Public Health, University of Padua, 35128 Padua, Italy; andrea.cozza@unipd.it; 9 Social Direction, Azienda ULSS 6 (Unità Locale Socio Sanitaria) “Euganea”, 35131 Padua, Italy; stefano.vianello@aulss6.veneto.it

**Keywords:** lower gastrointestinal endoscopy, waiting lists, multidisciplinary team, Homogeneous Waiting Groups

## Abstract

**Background:** Lower gastrointestinal endoscopy is a critical diagnostic and therapeutic procedure that is inherently resource-intensive. Following the COVID-19 pandemic, backlogs grew significantly, necessitating innovative management solutions. This study evaluates a multidisciplinary strategy integrating organizational interventions supported by a machine-learning-based semantic appropriateness assessment system to reduce pre-appointment waiting lists within the ULSS (Unità Locale Socio Sanitaria) 3 Serenissima health authority in the Venice metropolitan area. **Methods:** We retrospectively analyzed administrative and prescription data for adult patients undergoing lower gastrointestinal endoscopy from 2021 to 2024 across five hospitals. Evaluation metrics included the size and composition of the pre-appointment list (first vs. follow-up), service volumes, and prescription trends. Clinical appropriateness was assessed pre-procedure by adherence to RAO (Raggruppamenti di Attesa Omogenea, i.e., Homogeneous Waiting Groups) priority protocols and post-procedure by specialist reviews against guideline-based criteria in two samples from 2023 and 2024. **Results:** The pre-appointment list decreased significantly from 1, 542 in 2021 to only 14 in 2024. Procedural volume increased (+8% in 2023 vs. 2022), while overall prescriptions decreased, reflecting improved demand management. The provided-to-prescribed ratio rose from 64% in 2021 to 80% in 2024. Furthermore, RAO priority discordance declined, and inappropriate priority-class lower gastrointestinal endoscopy dropped from 26% in 2023 to 21% in 2024—a 5% absolute reduction. **Conclusions:** A combined strategy targeting both supply and demand—utilizing multidisciplinary governance, computerized decision-support management based on semantic analysis and RAO appropriateness assessment, structured pathways, and continuous audit—was associated with a marked reduction in waiting lists and improved appropriateness while simultaneously increasing service delivery. Expanding specialist–primary care consultation pathways, including teleconsultation, may further consolidate these improvements.

## 1. Introduction

Digestive endoscopy is a widely used, safe, and low-mortality method for diagnosing and treating gastrointestinal diseases, making it one of the most frequently provided outpatient services [1]. However, the SARS-CoV-2 pandemic severely disrupted healthcare delivery, sharply reducing outpatient volume and generating extensive waiting lists [2,3].

In Italy, when a service cannot be provided immediately, patients are placed on pre-appointment waiting lists managed through several strategies, including scheduling additional sessions, recall systems, and filling slot vacancies from cancellations [4]. According to regional guidelines in Veneto, these lists serve to take charge of the patient, guarantee the service within the prescribed priority timeframe, and contact users to finalize bookings [5,6].

Implementing and managing lower gastrointestinal endoscopy services presents several unique operational challenges [7,8,9]:

- Procedure Time: Lower gastrointestinal endoscopy often requires conscious sedation and continuous monitoring, taking two to three times longer than standard outpatient procedures, which delays list clearance.
- Staffing Requirements: Modern standards require at least three healthcare professionals per endoscopic session to manage the procedure, instrument reprocessing, and post-sedation recovery.
- Competing Resource Demands: Gastroenterology units must balance outpatient care with emergency procedures, inpatient investigations, and colorectal cancer screening—the latter of which demands dedicated pathways and exclusive resources.

Given these management complexities, this study analyzes pre-appointment lower gastrointestinal endoscopy services from 1 January 2021 to 31 December 2024 within the Unità Locale Socio-Sanitaria (ULSS 3) Serenissima of Venice, assessing the underlying critical issues and the effectiveness of corrective actions.

## 2. Materials and Methods

### 2.1. Study Population

As of 31 December 2022, the resident population in the area served by ULSS 3 Serenissima was approximately 611,434 inhabitants, 51.4% female and 48.6% male, across 23 municipalities. As of January 1, 2023, data show a demographic distribution by age group: children (0–13 years) about 10%, working-age adults (14–64) about 63%, people aged 65–74 about 12%, and those over 75 around 15%. A particularly notable feature is the share of older residents: over 26% are over 65 years old—well above the regional average of 24.1%.

Data extraction started in January 2021; however, annual comparisons were based on homogeneous September audits, with September 2021 representing the first complete operational assessment. For waiting-list analyses, the unit of analysis was the individual procedure (service) recorded within the pre-appointment list. A single referral could include more than one procedure code and therefore contribute more than once to service-volume analyses. The study was performed in the five main hospitals of the ULSS 3 Serenissima, i.e., Venezia (historical center, Santi Giovanni e Paolo; Mestre, Ospedale dell’Angelo; Chioggia; Dolo/Mirano). The participants were included in the study if they were adults (>18 years old) and undergoing a lower gastrointestinal endoscopy for any reason. In detail, in this study, only outpatient diagnostic referral was included. No other exclusion criteria were applied, in order to be representative of the general population living in our territory, except clinical contraindications to lower gastrointestinal endoscopy (e.g., anticoagulant use on the same day as the test). The same patient could contribute to only one referral, prescription or procedure during the study period to the analysis.

This study was conducted in accordance with the Declaration of Helsinki and received formal retrospective research approval for the secondary analysis of existing administrative data from the Ethics Committee (Comitato Etico Territoriale Area Centro-Est Veneto, protocol code 0066641, approved on 19 February 2026). Because the analyzed data had already been completely recorded for routine clinical and administrative healthcare delivery purposes between 2021 and 2024, individual patient informed consent was explicitly waived by the Ethics Committee due to the strictly retrospective nature of the study. To maintain data governance and privacy, administrative and prescription records were electronically extracted from the centralized healthcare network across the five participating hospitals using the primary analytics engine, Clinika. Procedures were isolated utilizing specific regional prescriptive catalog codes for lower gastrointestinal endoscopies.

### 2.2. Details of Semantic Engine Analysis System

To support prescription appropriateness assessment, the ULSS 3 health authority adopted Clinika VAP (Maps Healthcare, more details at: https://mapsgroup.eu/healthcare/, accessed on 30 April 2026), a computerized decision-support platform integrated within the waiting-list governance system.

Clinika VAP combines a semantic-analysis machine learning engine with a rule-based RAO evaluation system. The semantic engine analyzes free-text diagnostic queries using a medical ontology developed from Italian clinical language, while the rule-based component evaluates consistency between the reported indication and the assigned RAO priority category.

Input data included prescription records, diagnostic queries, priority classes, prescribing physician category, and administrative information. The system automatically identified non-concordant prescriptions, false first-access requests, and potentially inappropriate priority assignments.

The platform had previously undergone validation against specialist-reviewed test sets and was considered operational only after achieving at least 90% agreement with expert evaluations, according to the technical specifications of the system [10].

The data-cleaning and deduplication process focused heavily on administrative precision; automated algorithmic screening via Clinika was used to identify and filter out ‘False First-Accesses’ (misclassified follow-up appointments), thereby isolating a clean cohort of ‘Consistent Clinical Queries’. Records were linked and categorized according to the prescribing physician’s operational profile (General Practitioner vs. Specialist). Missing data fields were systematically flagged as ‘NAs’ and proved to be negligible, accounting for less than 0.4% of the total database entries across all evaluated years.

### 2.3. Lower Gastrointestinal Endoscopy

Lower gastrointestinal endoscopies were identified using the prescriptive codes from the regional catalog:

- 45.23 colonoscopy with flexible endoscope;
- 45.23.1 retrograde ileocolonoscopy;
- 45.24 recto-sigmoidoscopy with flexible endoscope. endoscopy of descending colon;
- 45.25 pancolonoscopy with biopsy, biopsy of nonspecific intestinal site bushing or washing for sample collection.

Patients subsequently referred to screening or surveillance programs were retained in the study analyses, and in that way every pre-appointment on the list had undergone lower gastrointestinal endoscopy. The transfer occurred only after completion of the procedures and therefore did not influence waiting-list reductions or service-volume estimates.

Again, by means of computerized data extraction, we went on to identify, for the prescription codes listed above, how much was prescribed by General Practitioners and how much by Specialist Doctors, in order to assess the volumes of services prescribed and provided and the appropriateness of the prescriptions themselves. We included in the category ‘others’ freelance physicians or those physicians not located in the Venice Metropolitan area.

The details of the main and secondary outcomes are reported in Supplementary Table S1.

### 2.4. Statistical Analysis

Statistical analysis was performed to evaluate temporal changes in lower gastrointestinal endoscopy service demand, provision, and appropriateness between 2021 and 2024. List sizes, service counts, prescription counts, and improvement of appropriateness were considered as outcomes of the study. Categorical variables, including the proportion of first versus follow-up visits and rates of RAO discordance, were compared across time points using the chi-square test for proportions. Percentage change over time was calculated to quantify reductions in pre-appointment services and increases in service provision. Appropriateness was assessed both pre-service, via RAO protocol adherence, and post-service, by calculating the proportion of procedures deemed unnecessary according to guideline-based criteria. Statistical significance was set at *p* < 0.05 for all comparisons, and analyses were conducted using IBM SPSS 26.0.

## 3. Results

**Figure 1:**
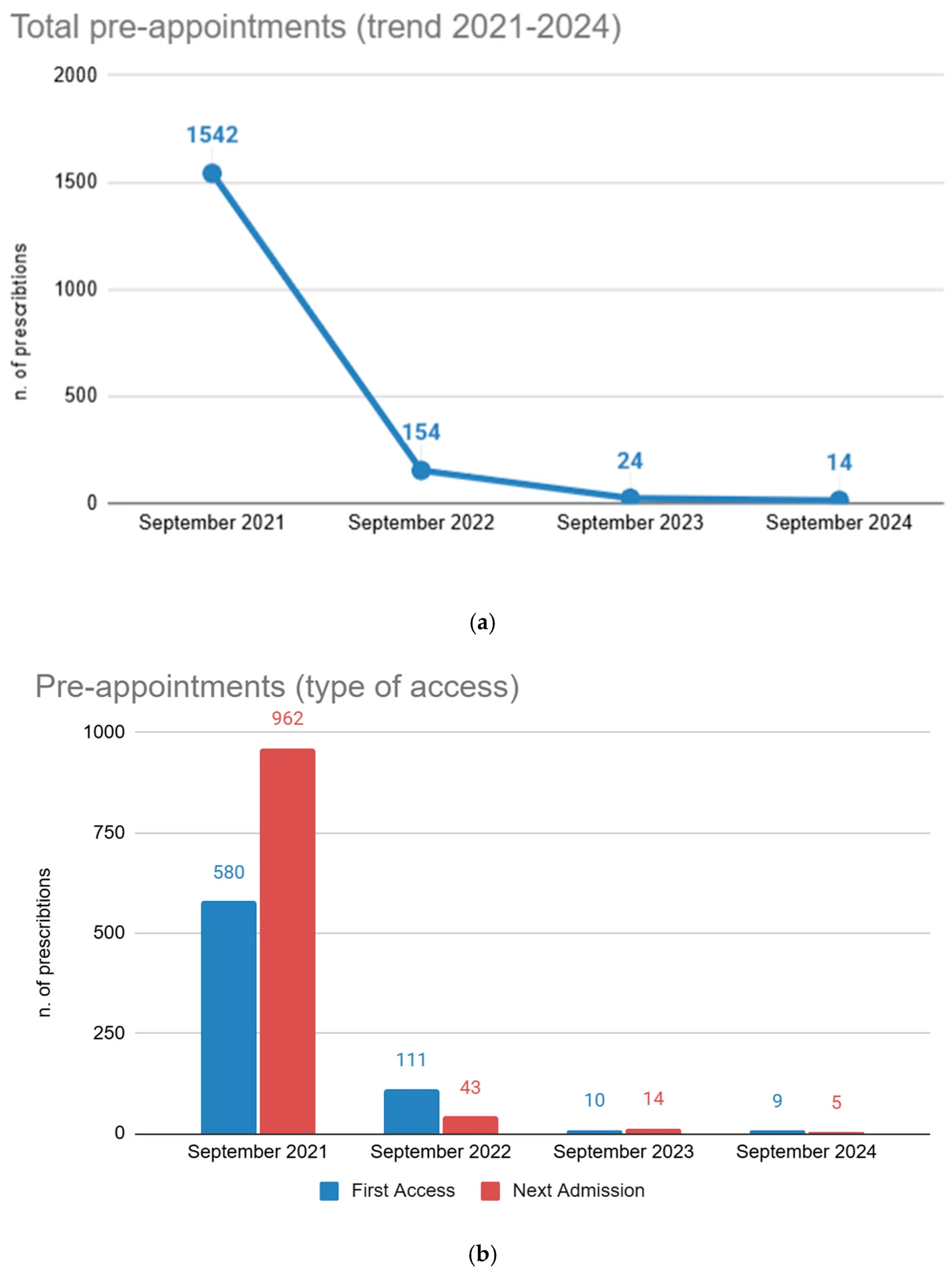
Active pre-appointment waiting-list entries (total in (**a**) and by type in (**b**)) recorded during homogeneous September audits between 2021 and 2024, in ULSS 3 Serenissima.

The dataset showed a substantial reduction in pre-appointment services from 2021 to 2024, as shown in Figure 1a. The total volume of appointments plummeted from 1542 in 2021 to just 154 in 2022, marking a significant statistical reduction of 90.1% compared to 2021. This downward trajectory continued with high consistency through 2023 and 2024, reaching a volume of only 14 total appointments in September 2024, marking a significant statistical reduction of 99.1% compared to 2021.

Figure 1b shows that the internal composition of the service underwent a statistically significant shift in patient mix. In September 2021, the workload was heavily weighted toward follow-up visits (62.3% of the total). By September 2024, this dynamic had inverted, with “First Access” visits representing 64.3% of the remaining marginal activity. Chi-square testing confirms that these proportional changes are significant (*p*-value < 0.05), indicating that the service not only shrank but also fundamentally altered its clinical focus. The combination of a near-total volume loss and a significant shift in service delivery models confirms a systemic transition in the pre-appointment protocol over the analyzed period.

**Figure 2:**
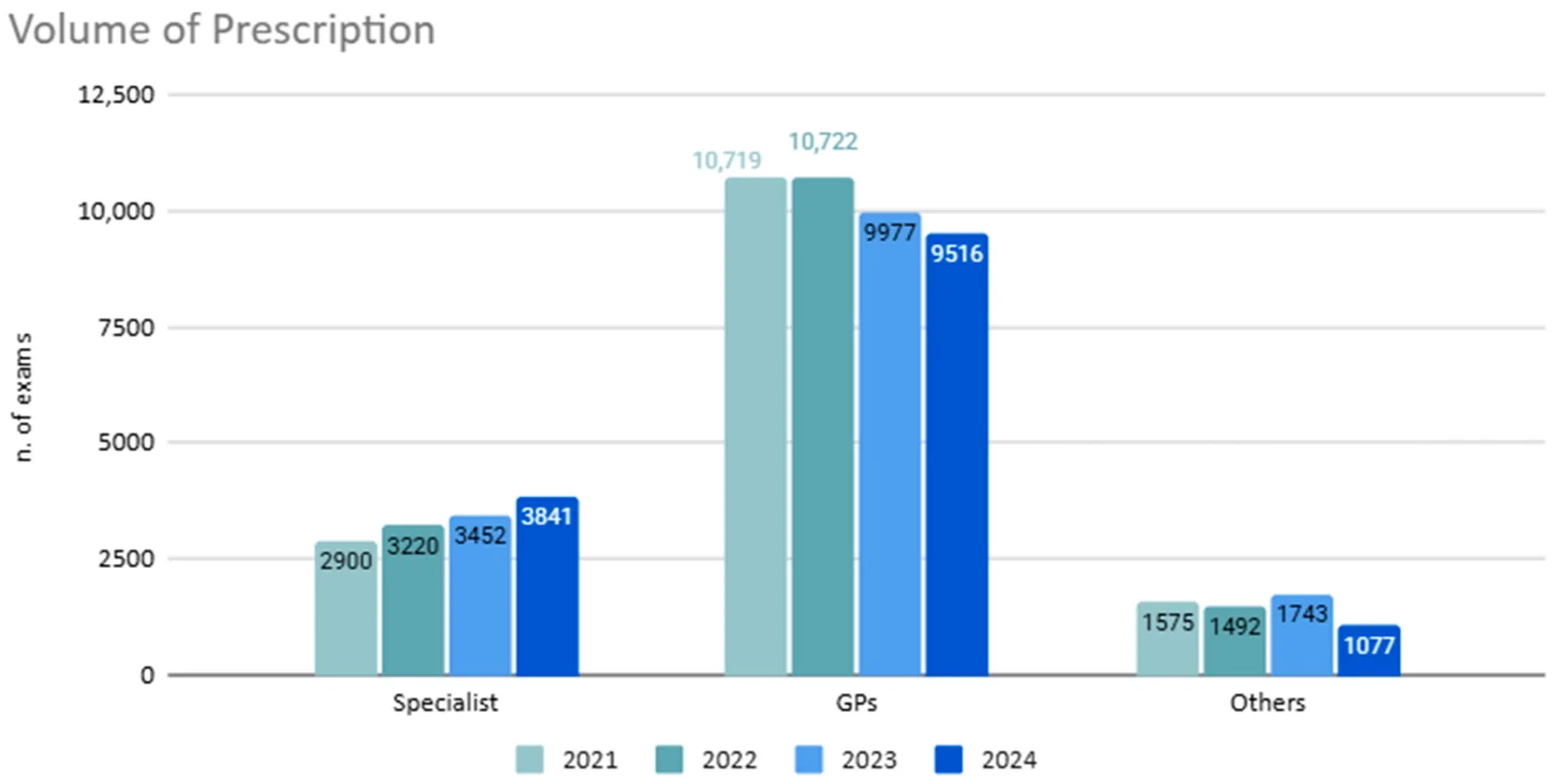
Number of exams prescribed between 2021 and 2024 in ULSS 3 Serenissima.

As shown in Figure 2, in 2024 the number of exams prescribed by the specialists accounted for 26.6% of the total prescriptions, with an increase, compared to 2021, of 32.4%. At the same time, the number of exams prescribed by General Practitioners in 2024 was 65.9% of the total, with a reduction of 11.2% compared to 2021.

**Figure 3:**
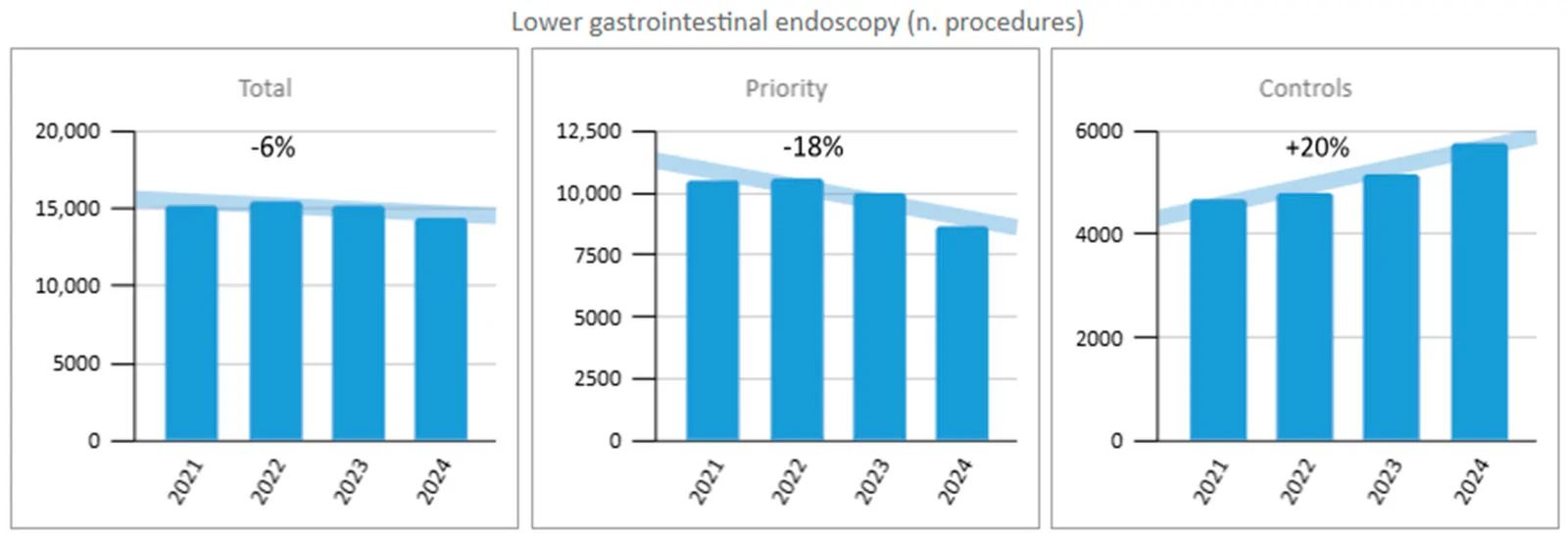
Changes in the rate of endoscopy exams prescribed between 2021 and 2024 in ULSS 3 Serenissima.

Shifting the focus to lower gastrointestinal endoscopy services as a whole shows a 5% reduction in prescriptions between 2021 and 2024, the result of the ULSS 3 Serenissima’s initiatives aimed at improving prescriptive appropriateness, particularly for an increase of 20% in controls, as graphically reported in Figure 3.

**Figure 4:**
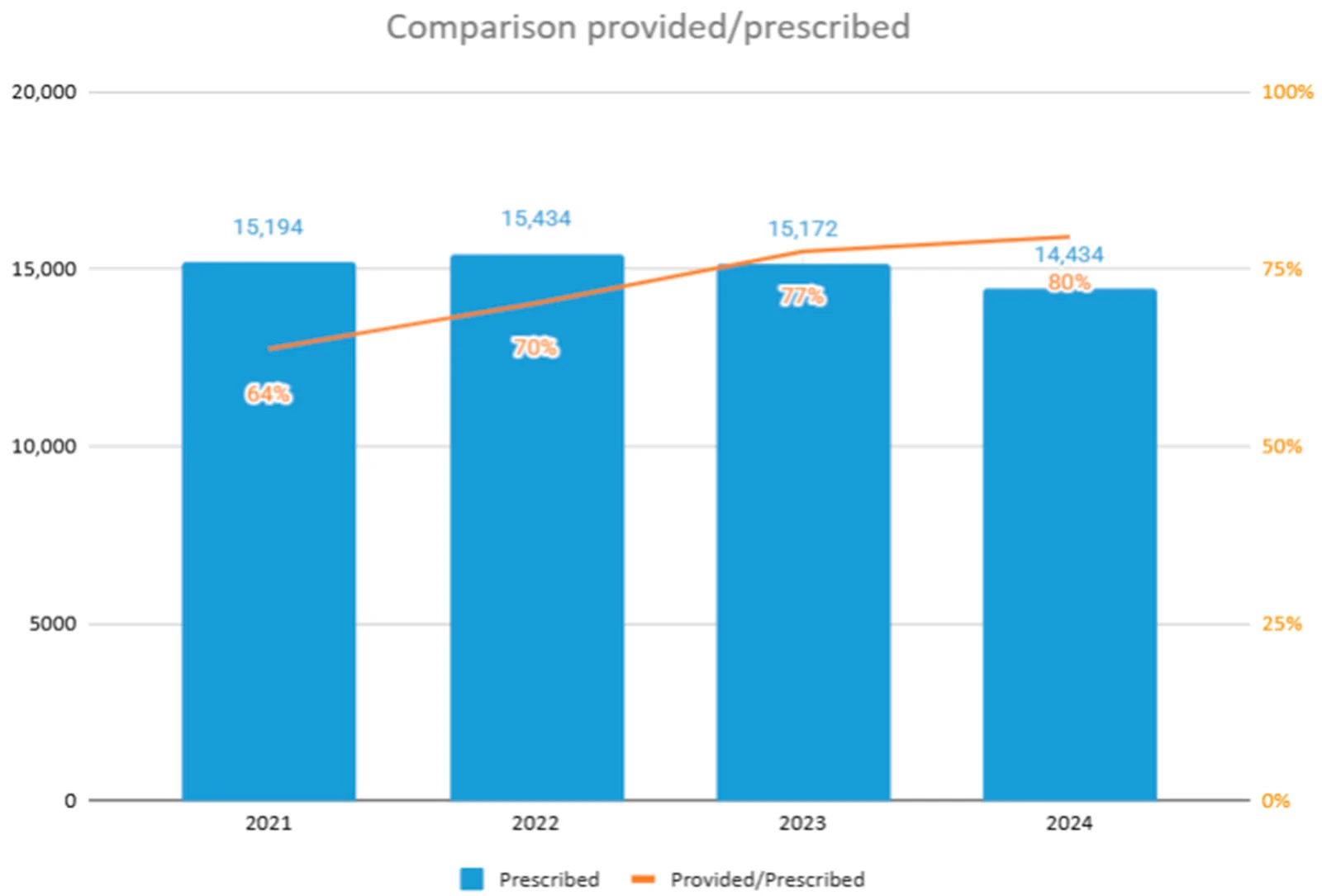
Comparison between services prescribed and provided between 2021 and 2024, with the provided-to-prescribed ratio, in ULSS 3 Serenissima.

Analyzing the ratio between services supplied and prescribed over the years, while there has been a reduction in prescriptions, there has been an increase in the services supplied, from 64% in 2021 to 80% in 2024, thus reducing the gap between prescribed and supplied (*p*-value < 0.05 for the trend), as reported in Figure 4.

**Figure 5:**
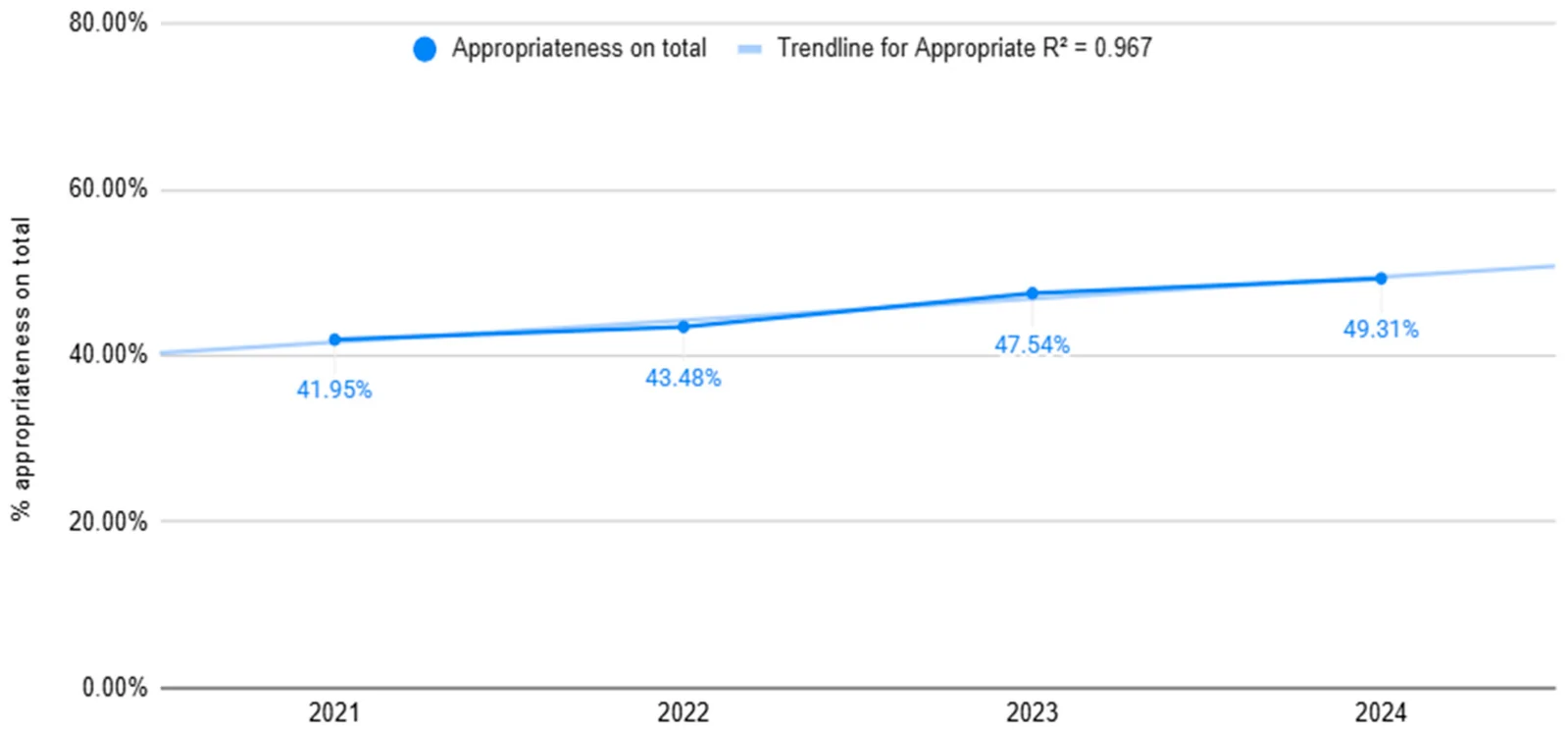
Analysis of clinical appropriateness and RAO concordance for lower gastrointestinal endoscopy within the ULSS 3 Serenissima (2021–2024). Source: Clinika 3.3.

Figure 5 shows the results of the analysis on prioritized colonoscopy lower gastrointestinal endoscopy referrals (years 2021–2024). RAO Concordance on Total Prescriptions rose steadily from 41.95% in 2021 to 49.31% in 2024; this trend reflects the effectiveness of the multidisciplinary strategy in filtering out “noise” and administrative errors, such as False First-Accesses, which saw a significant reduction over the four-year period.

An assessment of appropriateness after the services have been provided was also carried out, an analysis which has a very high value in providing an estimate of the percentage of services that are provided but not necessary.

As reported in Table 1, the specialists in endoscopy (F.B., S.A., F.C., S.D.B.) analyzed a sample of 903 services provided during the second half of 2023. It emerged that 26% of the services prescribed in priority class and provided were not appropriate, according to appropriateness criteria defined by guidelines and good medical practice reported in Hassan et al. 2020 [11], considering the indication for the examination by the prescribing physician and the anamnestic data collected from the patient prior to the procedure. The same analysis was performed during the period January–June 2024 on a sample of 963 services provided with evidence that 21% of the services prescribed in priority class and provided were not appropriate, highlighting an improvement in prescriptions by GPs.

## 4. Discussion

As of 30 September 2021, Azienda ULSS 3 Serenissima faced a backlog of 1537 gastrointestinal endoscopy patients awaiting appointments. To address this substantial volume, an internal analysis was conducted to identify the critical issues affecting the lower gastrointestinal endoscopy waiting list. Corrective actions were subsequent targeted at managing both supply (A) and demand (B) [12,13,14,15].

Our analysis and the supporting literature highlight four main factors driving inappropriate demand for lower gastrointestinal endoscopy:

1. Inappropriate Follow-up Timing: Guidelines dictate specific surveillance intervals for colorectal polyps based on risk stratification. However, waiting list data revealed a high frequency of premature or unnecessary follow-up prescriptions requested outside the structured clinical surveillance.
2. Out-of-Pathway Occult Blood Testing: While the fecal occult blood test (FOBT) is a free, structured screening tool for specific age groups (extended from 50 to 69 to 54–74 years in Veneto), it is frequently requested inappropriately through standard outpatient channels. This includes testing in patients on anticoagulant therapies, where a positive result is expected but still triggers a resource-consuming second-level assessment. This pattern aligns with over-prescription warnings highlighted by the Choosing Wisely Italy program [16].
3. Vague Familial Profiles: While clear guidelines govern surveillance for genetically determined pathologies or true familial risk [17], many pre-appointment requests were driven by generic, undocumented family history that lacked formal disease risk validation.
4. Conflation of Booking Channels: Screening (preventive) and diagnostic lower gastrointestinal endoscopies operate through parallel, distinct booking channels. Despite this, a significant portion of the diagnostic waiting list consisted of patients requesting procedures for screening or prevention purposes within the age bracket already covered by the official screening program.

To optimize the appropriateness of prescriptions and enhance the alignment between prescribing general practitioners (GPs) and providers, several organizational models and strategic actions were deployed [15]. First, to bridge educational gaps, the health authority hosted targeted conferences and meetings incentivized by Continuing Medical Education (CME) credits. This included widespread sharing of Choosing Wisely Italy documentation to foster clinical consensus on necessary medical practices. Second, the RAO (Raggruppamenti di Attesa Omogenei) model was integrated directly into GP prescription management software [18,19,20]. By providing real-time, evidence-based criteria based on patient severity, this tool helps standardize prescribing behavior and ensures equitable access within appropriate priority windows. Third, ULSS 3 Serenissima instituted mandatory quarterly reporting on GP prescription performance to maintain ongoing professional accountability and transparency. Fourth, in our experience, transferring post-diagnostic management to endoscopy specialists helps guarantee proper downstream care paths. This strategy includes deferring final lower gastrointestinal endoscopy reports until histological findings are fully reviewed to establish accurate surveillance timelines [20]. It also involves providing dedicated communication channels (shared emails and telephone lines) to facilitate rapid clinical consultation between GPs and endoscopists.

The findings of our paper must be considered within its limitations. First, the analysis was conducted retrospectively using administrative and prescription records, which may be prone to coding errors, incomplete data entry, or misclassification of indications. Second, we were not able to capture the information of those patients accessing private services instead of public ones since these services followed other administrative paths. Third, it is possible that some groups that could need lower gastrointestinal endoscopy, such as immigrant, homeless or very elderly patients, are not adequately represented. Third, the findings are based on a single health authority (ULSS 3 Serenissima) and may not be directly generalizable to other regions with different demographic profiles, healthcare structures, or resource availability. Finally, while the multidisciplinary interventions implemented within ULSS 3 Serenissima—including management supported by the Clinika VAP decision-support platform and the adoption of structured RAO priority protocols—were associated with improvements in service delivery and prescribing appropriateness, the observed reduction in waiting-list volume should not be attributed exclusively to these measures. Other factors, including regional policies promoting referral redirection, the progressive normalization of healthcare activities after the COVID-19 pandemic, potential underrepresentation of vulnerable populations, and unmeasured migration toward private healthcare providers, may also have contributed to the observed trends.

## 5. Conclusions

The implementation of targeted supply- and demand-management strategies within Azienda ULSS 3 Serenissima was associated with measurable improvements across all primary performance indicators. The intervention was linked with reduced overall waiting-list counts and the backlog of pre-appointment services. This was achieved through an increase in provided procedure volumes and optimized prescription volumes, which significantly enhanced the provided-to-prescribed ratio. Furthermore, the integration of standardized protocols resulted in a 5% improvement in assessed clinical appropriateness and a higher rate of RAO concordance among prescribing physicians, demonstrating the effectiveness of structured administrative and educational workflows in balancing endoscopic supply and demand.

## Figures and Tables

**Table 1:** Evaluation of the appropriateness of the exams completed and assessed by the endoscopy specialists.

Year	Number of Exams	Rate of Non-Appropriateness	Change
2023	903	26%	-
2024	963	21%	−5%

## Data Availability

Data are available upon request to the corresponding author due to constraints due to our local ethical committee.

## Supplementary Materials

The following supporting information can be downloaded at https://www.mdpi.com/article/10.3390/healthcare14162499/s1, Table S1. Operational Definitions, Outcome Classifications, and Administrative Derivations of Study Variables.

## Abbreviations

The following abbreviations are used in this manuscript:

AULSS 3/ULSS 3	(Azienda) Unità Locale Socio-Sanitaria 3
CME	Continuing Medical Education
FOBT	Fecal Occult Blood Test
GAS	Gastropack Access System
GPs	General Practitioners
RAO	Raggruppamenti di Attesa Omogenea (Homogeneous Waiting Groups)
