# Supplementary material for: A Multidisciplinary Strategy for Managing Lower Gastrointestinal Endoscopy Waiting Lists Within the ULSS 3 Serenissima Health Authority in the Venice Metropolitan Area
Source: Healthcare (Basel). 2026 Aug 12;14(16):2499. doi: 10.3390/healthcare14162499 (PMC13511986; doi:10.3390/healthcare14162499)
Supplement: Supplementary file 1 [file healthcare-14-02499-s001.zip › healthcare-4356558-supplementary.pdf]

Supplemental material S1. Operational Definitions, Outcome Classifications, and Administrative Derivations of Study Variables.

**Technical Term**	**Operational Definition**	**Outcome Classification**	**Derivation and Extraction Method (Clinika / Administrative System)**
Pre-appointment (pre-appuntamento)	A valid prescription who have been taken into charge by the health authority but is not yet scheduled in an active booking slot (waiting to be contacted for scheduling).	Primary Outcome	Prescription in an "unbooked" or "pending scheduling" status, extracted during homogeneous September audits (2021–2024).
Waiting list (Lista d'attesa)	The overall system queue, encompassing patients on the pre-appointment list.	Baseline Context / System Metric	Calculated as the sum of active pre-appointments in CUP (Centralized Booking Office) system
First access (Primo accesso)	An outpatient diagnostic procedure delivered to a patient who is studied for the first time and is not currently enrolled in a structured surveillance or follow-up pathway for the same pathology.	Primary Outcome (Case-mix metric)	Identified by the prescriptive administrative flag indicating "first visit/exam" and validated by the absence of follow-up keywords.
Next admission / Controls (Accesso successivo / Controlli)	Scheduled surveillance, follow-up, or control procedures (e.g., post-polypectomy surveillance).	Primary Outcome (Case-mix metric)	Identified by the presence of explicit follow-up keywords (e.g., "controllo", "follow-up", "sorveglianza") in the clinical query.
Provided / Supplied (Erogato / Fornito)	Lower gastrointestinal endoscopy procedures that were successfully completed, reported, and billed by the healthcare facilities.	Secondary Outcome	Extracted from hospital outpatient discharge records (SDA) using regional catalog codes: 45.23, 45.23.1, 45.24, 45.25.
Priority prescription (Prescrizione prioritarizzata)	A medical prescription carrying an explicit clinical urgency class (U: Immediate, B: 10 days, D: 30 days, P: 60-90	Baseline Input Variable	Extracted directly from the dematerialized prescription flow generated by GPs and

**Technical Term**	**Operational Definition**	**Outcome Classification**	**Derivation and Extraction Method (Clinika / Administrative System)**
	days) based on the regional RAO framework.		specialists.
False first-access (Falso primo accesso)	A prescription administratively flagged by the prescriber as a "First Access" (often to secure shorter wait times) but containing a clinical query that describes a follow-up or surveillance scenario.	Primary Outcome (Administrative appropriateness)	Identified by the Clinika VAP semantic engine via a mismatch between the administrative "first-access" flag and the clinical text.
Consistent clinical queries (Quesiti clinici coerenti)	The cleaned subset of prescriptions obtained after filtering out records with missing data (NAs) and administrative errors (i.e., "False First-Accesses").	Denominator for Analysis	Derived algorithmically by subtracting "NAs" and "False First-Accesses" from the total pool of prescriptions.
RAO concordance (Concordanza RAO)	The clinical-administrative agreement between the clinical indication described in the query text and the specific urgency class assigned.	Primary Outcome (Prescriptive appropriateness)	Calculated as the percentage of Consistent Clinical Queries where the assigned priority matches the clinical criteria of the RAO guidelines.
Appropriateness (Appropriatezza)	The clinical necessity and utility of the procedure. Evaluated ex-ante (adherence to RAO priority criteria) and ex-post (clinical utility based on medical records).	Primary Outcome	Ex-ante: semantic analysis from Clinika VAP.<br><br>Ex-post: retrospective specialist audit of clinical indicators on random patient samples (2023 vs. 2024).
